# Transcriptional Regulation of Macrophages Polarization by MicroRNAs

**DOI:** 10.3389/fimmu.2018.01175

**Published:** 2018-05-28

**Authors:** Heng Li, Ting Jiang, Meng-Qi Li, Xi-Long Zheng, Guo-Jun Zhao

**Affiliations:** ^1^The Clinic Medical College, Guilin Medical University, Guilin, Guangxi, China; ^2^Department of Practice Educational, Office of Academic Affairs, Guilin Medical University, Guilin, Guangxi, China; ^3^Department of Histology and Embryology, Guilin Medical University, Guilin, Guangxi, China; ^4^Department of Biochemistry and Molecular Biology, The Libin Cardiovascular Institute of Alberta, The University of Calgary, Health Sciences Center, Calgary, AB, Canada; ^5^Key Laboratory of Molecular Targets and Clinical Pharmacology, School of Pharmaceutical Sciences, Guangzhou Medical University, Guangzhou, Guangdong, China

**Keywords:** microRNAs, transcription factors, macrophages polarization, tumor, immunity

## Abstract

Diversity and plasticity are the hallmarks of cells from the monocyte–macrophage lineage. Macrophages undergo classical M1 or alternative M2 activation in response to the microenvironment signals. Several transcription factors, such as peroxisome proliferator-activated receptors, signal transducers and activators of transcription, CCAAT-enhancer-binding proteins, interferon regulatory factors, Kruppel-like factors, GATA binding protein 3, nuclear transcription factor-κB, and c-MYC, were found to promote the expression of specific genes, which dictate the functional polarization of macrophages. Importantly, these transcription factors can be regulated by microRNAs (miRNAs), a group of small non-coding RNAs, which regulate gene expression through translation repression or mRNA degradation. Recent studies have also revealed that miRNAs control macrophage polarization by regulating transcription factors in response to the microenvironment signals. This review will summarize recent progress of miRNAs in the transcriptional regulation of macrophage polarization and provide the insights into the development of macrophage-centered diagnostic and therapeutic strategies.

## Introduction

Monocyte-macrophage lineage cells exist in various tissues in the body and play an important role in homeostasis, cancer, wound healing, and immune response (1, 2). Macrophages are derived from bone marrow-derived monocytic cells *via* a process of differentiation (3, 4). Circulating monocytes migrate into the majority of tissues in the body, where they differentiate into functionally distinct mature macrophages (4). Besides, it was also described that tissue-resident macrophages originate from yolk-sac-derived erythro-myeloid progenitors (5, 6). Adult Langerhans cells are derived predominantly from embryonic fetal liver monocytes with a minor contribution of yolk sac-derived macrophages (7). Monocyte–macrophage lineage cells are featured by functional diversity and plasticity. The classically activated M1 and alternatively M2 macrophages represent two extremes of a dynamic changing state of macrophage activation. In response to the microenvironment signals, macrophages can rapidly switch from one polarization state to the other (8, 9). It is known that the dynamic change of macrophage activation is directed by the activation of specific transcription factors, such as peroxisome proliferator-activated receptors (PPARs), signal transducers and activators of transcription (STATs), CCAAT-enhancer-binding proteins (C/EBPs), interferon regulatory factor (IRF), Kruppel-like factors (KLFs), GATA binding protein (GATA) 3, c-MYC, and nuclear transcription factor-κB (NF-κB) (3, 4).

M1 macrophages, also known as classically activated macrophages, can be activated by toll-like receptor (TLR) ligands, such as lipopolysaccharides (LPS) or interferon-γ (IFN-γ). M1 macrophages are characterized by high antigen presentation, high expression of pro-inflammatory cytokines [e.g., interleukin (IL)-12, IL-23, and tumor necrosis factor-α (TNF-α)], and high production of reactive nitrogen intermediates and reactive oxygen intermediates. M1 macrophages are supposedly associated with inflammatory, microbicidal, and tumoricidal activities (10–12). M2 macrophages, also called alternatively activated macrophages, can be further subdivided into subsets called M2a, M2b, M2c, and M2d. The Th2 cytokines such as IL-4 and IL-13 bind to IL-4 and IL-13 receptors to induce the formation of M2a macrophages, whereas M2b macrophages are induced by immunoglobulin complexes in combination with TLR agonists, and M2c macrophages are induced by IL-10, transforming growth factor β (TGF-β), or glucocorticoids (13–15). Within the tumor, macrophages are a major stromal component, where they are commonly termed tumor-associated macrophages (TAMs). TAMs exhibit functions similar to those of M2 macrophages and can be characterized as the M2d subtype (16). M2 macrophages are characterized by an IL-12^low^IL-10^high^IL-1decoyR^high^IL-1RA^high^ phenotype with efficient phagocytic activity, high expression of mannose and galactose receptors, high levels of scavenging molecules, and high expression of specific markers of alternative activation, such as arginase-1 (Arg-1), found in inflammatory zone 1 (Fizz1) and chitinase-3-like protein 3 (Ym1). M2 macrophages are responsible for tuning inflammatory responses, adaptive immunity, parasite infection, tissue remodeling and repair, scavenge debris, and promoting angiogenesis and tumor progression (17–20). Transcription factors are the key molecules to determine the expression of specific genes and closely regulated by various signaling molecules in macrophages. The transcriptional regulation of macrophage polarization has been the focus of numerous recent studies. For example, STAT1, C/EBP-α, C/EBP-δ, IRF9, KLF6, and NF-κB are important transcription factors involved in polarization of M1 macrophage, whereas PPARs, STAT3, STAT6, C/EBP-β, IRF4, KLF4, GATA3, and c-MYC are associated with M2 macrophage polarization (21–24).

Transcription factors control the transcription rates to regulate the amounts of gene products, but transcription factors themselves are also regulated. There are several ways that the activities of transcription factors are regulated. Like all proteins, transcription factors are transcribed from a gene to RNA, which is then translated into protein. Any of these steps involving transcription and translation can be regulated to affect the production of transcription factors. Many transcription factors are located in the cytoplasm before activation and undergo nuclear translocation in response to the appropriate signals, such as NF-κB that must translocate to the nucleus before activating target gene transcription (25). Some transcription factors, such as STAT proteins, must be phosphorylated before they can bind DNA (26). A few of transcription factors should interact with other transcription factors before activated, for example, LXR and RXR must form heterodimer before binding to specific DNA sequences called LXR-responsive elements in the target genes (27).

**Figure 1:**
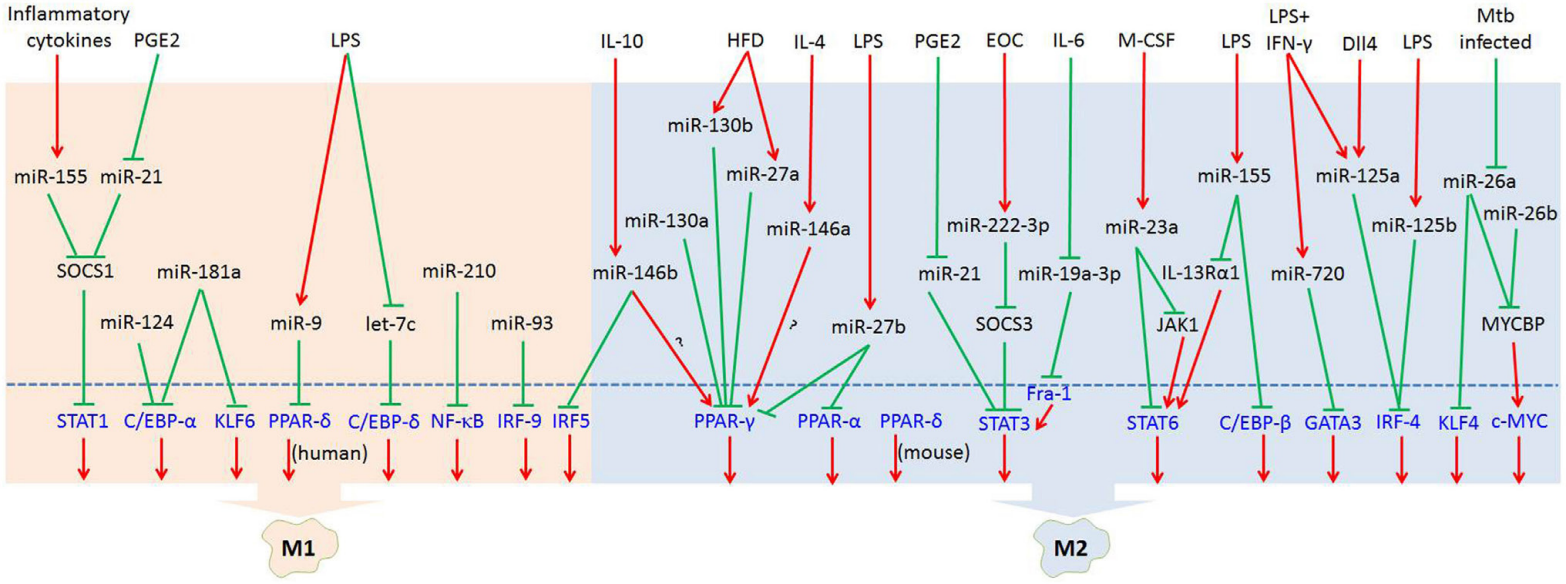
MicroRNAs (miRNAs) regulate macrophage polarization by targeting transcription factors in response to the microenvironment signals. In response to pathogen- and tissue-derived stimuli, miRNAs can control macrophage polarization by regulating transcription factors. Abbreviations: PGE2, prostaglandin E2; LPS, lipopolysaccharides; HFD, high-fat diet; IL-4, interleukin-4; EOC, epithelial ovarian cancer; IL-6, interleukin-6; M-CSF, macrophage colony-stimulating factor; IFN-γ, interferon-γ; Dll4, delta-like ligand 4; Mtb, *Mycobacterium tuberculosis*; SOCS1, suppressor of cytokine signaling 1; SOCS3, suppressor of cytokine signaling 3; JAK1, Janus kinase 1; IL-13Rα1, interleukin 13 receptor α1. 
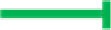
: inhibit; 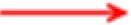: promote.

MicroRNAs (miRNAs) are endogenous small (20–22 nucleotides long) non-coding RNAs that induce inhibition of target gene expression within metazoan cells by binding to direct complementary sequences in the 3′ untranslated region (3′UTR) of mRNAs to target them for translational repression or degradation (28). miRNA regulation is characterized by its active participation in and strict control of the negative feedback loop to confer significant influences on a biological pathway (29). The roles of miRNAs in the regulation of macrophage polarization are largely unknown, and only several miRNAs are known to be involved in the regulation of macrophage polarization (30). Recent studies have shown that some miRNAs participate in macrophage polarization by regulating transcription factors. Hence, in this review, we focused on recent progress regarding the crucial roles of miRNAs in the regulation of macrophage polarization *via* modulating transcription factors (Figure 1; Table 1) and tried to provide a better understanding of the biological functions of miRNAs in macrophage polarization.

**Table 1:** MicroRNAs (miRNAs) control macrophage polarization by regulating transcription factors.

miRNAs	Targets	Pathways	Function	Cell types	Regulation of macrophage phenotype	Reference
miR-9	PPAR-δ	Directly	Suppresses inflammatory responses	PBMCs, HEK293	Suppresses M1	(37)
miR-27a	PPAR-γ	Directly	Promotes inflammatory responses	Raw264.7, 3T3-L1	Promotes M1, suppresses M2	(40)
miR-27b	PPAR-γ	Directly	Promotes inflammatory responses	THP-1, PBMCs	Promotes M1, suppresses M2	(43)
	PPAR-α	Directly	Promotes inflammatory responses	HuH7, HepG2	Promotes M1, suppresses M2	(44)
miR-130a	PPAR-γ	Directly	Promotes inflammatory responses	THP-1	Promotes M1, suppresses M2	(45)
miR-130b	PPAR-γ	Directly	Promotes inflammatory responses	Peritoneal macrophages (PMs)	Promotes M1, suppresses M2	(46)
miR-146a	?	PPAR-γ	Suppresses inflammatory responses	RAW264.7	Suppresses M1, promotes M2	(53)
miR-146b	?	PPAR-γ	Suppresses inflammatory responses	hMSCs, vHPA	Suppresses M1, promotes M2	(54)
	IRF5	Directly	Suppresses inflammatory responses	BMDMs	Suppresses M1, promotes M2	(100)
miR-155	SOCS1	STAT1	Promotes inflammatory responses	PBMCs, microglia, and HRPE	Promotes M1	(62, 63)
	IL-13Rα1	STAT6	Promotes inflammatory responses	THP-1, PBMCs, and HeLa	Suppresses M2	(61, 64)
	C/EBP-β	Directly	Suppresses tumor progression and metastasis	SK-Hep-1, HepG2, HeLa, THP-1, U251, and 95D	Suppresses M2	(84)
miR-21	STAT3	Directly	Promotes inflammatory responses	PMs	Suppresses M2	(66)
	SOCS1	STAT1	Promotes inflammatory responses	PMs	Promotes M1	(66)
miR-222-3p	SOCS3	STAT3	Promotes tumor progression and metastasis	Skov3, A2780, and U937	Promotes M2	(69)
miR-19a-3p	Fra-1	STAT3	Suppresses tumor progression and metastasis	4T1, 4T07, RAW264.7, and U937	Suppresses M2	(70)
miR-23a	JAK1	STAT6	Suppresses tumor progression and metastasis	RAW264.7, PMs, and BMDMs	Suppresses M2	(71)
miR-124	C/EBP-α	Directly	Suppresses inflammatory responses	BMDMs, microglia	Suppresses M1, promotes M2	(81, 82)
miR-181a	C/EBP-α	Directly	Promotes tumor progression and metastasis	THP-1, RAW264.7, MCF-7, OVCAR3, and HCT116	Suppresses M1, promotes M2	(83)
	KLF6	Directly	Promotes tumor progression and metastasis	THP-1, RAW264.7, MCF-7, OVCAR3, and HCT116	Suppresses M1, promotes M2	(83)
let-7c	C/EBP-δ	Directly	Suppresses inflammatory responses	GM-BMM, M-BMM	Suppresses M1, promotes M2	(86)
miR-125a	IRF4	Directly	Suppresses tumor progression and metastasis	BMDMs	Promotes M1, suppresses M2	(98)
miR-125b	IRF4	Directly	Suppresses tumor progression and metastasis	293T, RAW264.7, and BMMs	Promotes M1, suppresses M2	(99)
miR-93	IRF9	IRG1	Suppresses inflammatory responses	BMDMs	Suppresses M1, promotes M2	(96)
miR-26a	KLF4 MYCBP	Directly c-MYC	Promotes inflammatory responses Suppresses tumor progression and metastasis	RAW264.7, HEK293, EC109, KYSE450, and KYSE150	Promotes M1, suppresses M2 Suppresses M2	(104) (111, 113)
miR-26b	MYCBP	c-MYC	Suppresses tumor progression and metastasis	EC109, KYSE450, and KYSE150	Suppresses M2	(111, 113)
miR-720	GATA3	Directly	Suppresses tumor progression and metastasis	THP-1, MDA-MB-231	Suppresses M2	(105)
miR-210	NF-κB	Directly	Suppresses inflammatory responses	RAW264.7, HEK293	Suppresses M1	(109, 110)

*?, uncertain*.

## miRNAs Regulate Macrophage Polarization through Transcription Factors

### Peroxisome Proliferator-Activated Receptors

Peroxisome proliferator-activated receptors are a group of transcriptional factors belonging to the ligand-activated nuclear hormone receptor superfamily, including PPAR-α, PPAR-δ, and PPAR-γ (21, 31). PPARs have been shown to transcriptionally regulate the activation of macrophages in health and disease states, including cardiovascular disease, obesity, insulin resistance, and Chagas’ disease (31, 32). Penas et al. showed that PPAR-α ameliorates macrophage inflammatory responses and regulates the changes of the phenotype of peritoneal macrophages isolated from *T. cruzi*-infected mice (31). PPAR-α induces M2 macrophage polarization and enhances the expression of M2 markers (Arg-1, YM-1, Mrc1, and TGF-β) through inhibiting the expression of nitric oxide synthase 2 (NOS2) and pro-inflammatory cytokines in *T. cruzi*-infected mice (31). Kang et al. found that the deletion of the myeloid PPAR-δ gene results in adipocyte dysfunction, insulin resistance, and hepatosteatosis (33). IL-13 can upregulate the expression of PPAR-δ to induce the polarization of adipose tissue-resident macrophages toward the alternatively activated. Also, myeloid-specific PPAR-δ^−/−^ mice fed high-fat diet (HFD) showed increased M1 and decreased M2 markers in white adipose tissue and liver (33). PPAR-γ expression levels were positively correlated with the expression of M2 markers (Mrc1, AMAC1, and IL-10) in human carotid atherosclerotic lesions (34). Furthermore, PPAR-γ controls the inflammatory response by negatively interfering with pro-inflammatory signaling pathways, such as AP-1, NF-κB, or STAT3 in activated M1 macrophages (34).

The important roles of miR-9 in innate immune and anti-inflammatory response have been described (35, 36). miR-9 overexpression was reported to inhibit inflammation in rat mesenteric lymphatic endothelial cells (35). Recently, miR-9 was found to regulate PPAR-δ expression in human monocytes during the inflammatory response (37). It is believed that PPAR-δ can induce a phenotype switch from M1 to M2 macrophages in mice. However, Bouhlel et al. showed that activation of PPAR-δ does not promote human monocyte differentiation into anti-inflammatory M2 macrophages (38). In addition, Thulin et al. demonstrated that activation of PPAR-δ is of importance in M1 pro-inflammatory macrophages in humans (37). These studies may reflect differences between mouse and human macrophages in terms of the function of PPAR-δ. Bioinformatic analysis revealed that miR-9 can specifically bind to the 3′UTR of the PPAR-δ using a luciferase reporter construct. The expression of PPAR-δ mRNA is also suppressed by miR-9 in monocytes after treatment with pro-inflammatory agent LPS. Furthermore, inhibition of miR-9 was found to upregulate the mRNA expression of PPAR-δ in human primary monocytes (37). Thus, these results suggest that miR-9 inhibits macrophage inflammatory response *via* modulating the expression of PPAR-δ.

The miR-27 family is composed of two isoforms, miR-27a and miR-27b. Several studies demonstrated that miR-27 plays an important role in inflammation-related diseases, such as atherosclerosis and obesity (39–42). A recent study by Yao et al. revealed that miR-27a triggers an inflammatory response *via* promoting M1 macrophage polarization in obesity-induced insulin resistance in mice (40). They demonstrated that upregulation of miR-27a could suppress the expression of PPAR-γ with M1 polarization and increase the expression of p-NF-κB with degradation of IκBα in mice fed HFD. Jennewein et al. showed that the M1 activator LPS upregulates miR-27b to decrease the expression of PPAR-γ in M1 macrophages. Furthermore, inhibition of miR-27b impairs the ability of LPS to reduce the PPAR-γ mRNA half-life (43). Overexpression and inhibition of miR-27b in HuH7 cells significantly decreased and increased the PPAR-α levels, respectively (44). Thus, PPAR-α may be another transcription factor regulated by miR-27b, which is involved in the regulation of macrophage polarization. Taken together, miR-27 may serve as a crucial molecular regulator in the transcriptional regulation of macrophage polarization and may also be a potential therapeutic target for inflammatory diseases.

Low expression of miR-130a is closely associated with the progression and metastasis of non-small cell lung cancer (NSCLC) (45). It was found that miR-130a expression is inversely correlated with PPAR-γ and CD163 in NSCLC tissues. Furthermore, the expression level of miR-130a in M1 macrophage was much higher than that in M2 macrophages. Also, the flow cytometric analysis showed that transfection of macrophages with miR-130a mimic significantly enhanced the expression of CD80, iNOS, and TNF-α (M1 marker) in M1 macrophages but suppressed the expression of CD206, IL-10, and CCL22 (M2 marker) in M2 macrophages. Since the bioinformatic analysis demonstrated that PPAR-γ is a potential target of miR-130a, miR-130a may be involved in the regulation of macrophage polarization *via* modulating PPAR-γ (45). In the further study to elucidate the molecular mechanism of miR-130a involvement in the regulation of macrophage polarization, Lin et al. demonstrated that miR-130a skews polarization of human monocyte-derived macrophages from an M2 toward an M1 phenotype through targeting PPAR-γ 3′UTR for repression (45). In addition to miR-130a, miR-130b was found to promote adipose tissue inflammation and insulin resistance in diabetic mice *via* regulating macrophage polarization (46). Similar to the molecular mechanism of miR-130a-mediated M1/M2 macrophage polarization, miR-130b also skews their polarization toward an M1 phenotype *via* repression of PPAR-γ (46).

The miR-146 family comprises miR-146a and miR-146b, which promote tumor growth as well as exert a pro-inflammatory role in many diseases, such as Alzheimer, acute lung injury, coronary artery disease, and the impairment of diabetic wound healing (47–51). miR-146a/b has also been identified as negative regulators of TLR4 signaling (48, 52). TLR4 signaling plays an essential role in the regulation of M1 macrophage polarization. Also, Huang et al. found that miR-146a can enhance the activation of PPAR-γ to promote M2 macrophage polarization. The authors also demonstrated that overexpression of miR-146a significantly increases the expression of PPAR-γ, whereas interference of miR-146a decreases its expression at both protein and mRNA levels (53). Although PPAR-γ was involved in the miR-146a-mediated M2 macrophage polarization, the specific mechanism underlying miR-146a regulation of PPAR-γ remains unknown. Further research will be needed to explain how miR-146a works through PPAR-γ to regulate macrophage polarization. Besides miR-146a, miR-146b can significantly increase the expression of the transcription factor PPAR-γ (54), suggesting that miR-146b may enhance the activation of M2 macrophage *via* promoting PPAR-γ expression.

### Signal Transducers and Activators of Transcription

The members of the STAT protein family are the key transcription factors that mediate macrophage M1/M2 polarization. It has been demonstrated that activation of STAT1 promotes an inflammatory response in various diseases, such as atherosclerosis and inflammatory bowel disease (55, 56). STAT1 is the important mediator of M1 macrophage polarization induced by IFN-γ, which can be derived from innate lymphocytes or Th1 cells (22). IFN-γ ligand binding to its receptor induces Janus kinase 1/2-mediated tyrosine phosphorylation and subsequent dimerization of STAT1, which binds as a homodimer to cis elements known as IFN-γ-activated sites in the promoter of target M1 signature genes [e.g., NOS2, IL-12, and class II transactivator (CIITA)] (22). Activation of STAT3 plays a crucial role in the progression of human epithelial ovarian cancer by regulating macrophage polarization in the tumor microenvironment (57). IL-10 and IL-6 induce STAT3-mediated expression of genes (IL-10, TGF-β1, and Mrc1) associated with an M2-like phenotype. It has been reported that STAT6^−/−^ eosinophils are unable to migrate to the lung during allergic airway inflammation (58). STAT6 is the key transcription factor in IL-4- or IL-13-mediated M2 macrophage polarization. STAT6 was found to activate transcription of genes typical of M2 polarization, such as Mrc1, Retnlα, Fizz1, Chi3l3, and Ym1 (8). Also, STAT-mediated activation of macrophages is modulated by members of the SOCS family. For instance, IFN-γ stimulation upregulates suppressor of cytokine signaling 1 (SOCS1) and suppressor of cytokine signaling 3 (SOCS3), and, in turn, inhibit the action of STAT1 and STAT3, respectively (8, 59).

miR-155 is a multi-functional miRNA known to play diverse roles in inflammation and immunity. For instance, miR-155 promotes M1 macrophage polarization and exerts an antibacterial role in macrophages from Akt1^−/−^ mice (60). miR-155 is also a crucial molecule for the fine-tuning of allergic disease and asthma (61). Xu et al. revealed a key role for the miR-155/SOCS1 axis in defining PI3-K/Akt1-mediated M1 skewing *in vitro* and *in vivo* (60). Another study by Moore et al. showed that miR-155 mimic can significantly increase both pro-inflammatory cytokine and surface molecule expression by decreasing the expression of SOCS1 (62). Furthermore, Kutty et al. showed that the increase in miR-155 expression by the inflammatory cytokines was associated with an increase in STAT1 activation, as well as an increase in protein binding to putative STAT1 binding elements present in the miR-155 gene promoter region (63). Given that SOCS1 is a negative regulator of STAT1 in the regulation of M2 macrophage polarization. These findings suggest that the role of miR-155 in macrophage polarization is through regulation of the SOCS1/STAT1 pathway. In addition, Martinez-Nunez et al. demonstrated that miR-155 directly targets interleukin 13 receptor α1 (IL-13Rα1) and decreases the levels of IL-13Rα1 protein, resulting in decreased activation of STAT6 in human macrophages (61). By targeting IL-13Rα1 and regulating the STAT6 cascade, miR-155 may trigger pro-inflammatory immune responses in asthma and allergic disease (61). What is more, silencing miR-155 has a suppressive role in M1 macrophage polarization and drives macrophage polarization toward the M2 phenotype by increasing the phosphorylation of STAT6 (64).

It was reported that miR-21 induces inflammatory responses in macrophages by binding to murine TLR7 and human TLR8, and then triggers a TLR-mediated prometastatic inflammatory response, likely leading to tumor growth and metastasis (65). More recently, miR-21 was found to play a vital role in driving M1 and inhibiting M2 peritoneal macrophage polarization. Wang et al. showed that the M1 markers, such as TNF-α, IL-6, IL-1β, and IL-12p40, were either not expressed or expressed at lower levels, whereas M2 markers, such as IL-10, RETNL-α, Arg-1, and Chi3 l3 were elevated in miR-21^−/−^ macrophages when compared with those in wild-type (WT) macrophages (66). The authors also demonstrated that miR-21 can control macrophage polarization *via* regulating the expression of STAT3 and SOCS1. miR-21 mimic inhibited STAT3 and SOCS1 expression while the antagomir enhanced STAT3 and SOCS1 expression in WT macrophages. Furthermore, the miR-21 mimic can directly target STAT3 3′UTR sequences (66). However, some studies showed that miR-21 inhibits the pro-inflammatory macrophage polarization and enhances the anti-inflammatory macrophage phenotype in other macrophage cell lines (67, 68). These discrepancies may be due to the fact that miR-21 might exert different effects on macrophage polarization depending on cell types and *in vivo* microenvironment.

A recent report by Xiang et al. demonstrated that miR-222-3p promotes M2 macrophage polarization *in vitro* and *in vivo*, which can facilitate the progression and metastasis of epithelial ovarian cancer (69). The authors showed that miR-222-3p can suppress the expression of SOCS3 *via* targeting its 3′UTR. SOCS3 has been identified as a negative regulator of STAT3 in controlling M1 macrophage polarization. Furthermore, the STAT3 pathway was either activated or inhibited when transfected with the miR-222-3p mimic and the inhibitor in macrophages, respectively (69). Thus, these data suggest that miR-222-3p plays an important role in the polarization of tumor-promoting M2 macrophages by modulating the SOCS3/STAT3 pathway.

miR-19a-3p was reported to suppress the growth and metastasis of breast tumor by inhibiting macrophage skew to M2 phenotype *via* downregulating Fra-1 (70). Further study showed that transfection with miR-19a-3p mimic or inhibitor results in inhibition or stimulation of the expressions of the Fra-1 downstream genes pSTAT3 and STAT3, respectively (70). These studies suggest that miR-19a-3p plays an important role in the regulation of macrophage polarization by regulating the expression of the Fra-1/STAT3 pathway.

Recently, Ma et al. found that miR-23a exerts an anti-tumor effect by inhibiting M2 macrophage polarization (71). The underlying mechanism is that STAT6 occupies the miR-23a promoter and activates its transcription in IL-4-stimulated macrophages. Then, miR-23a, in turn, inhibits the Janus kinase 1 (JAK1)/STAT6 pathway and reduces the production of M2 phenotype cytokines by directly targeting JAK1 and STAT6 (71). Notably, STAT3, but not STAT1, also binds to the miR-23a cluster promoter, suggesting that STAT3 may be involved in the miR-23a-mediated inhibition of M2 macrophage polarization (71).

### CCAAT-Enhancer-Binding Proteins

The C/EBP family, a member of bZIP (basic region leucine zipper) transcription factors, plays crucial roles in myeloid development and macrophage activation (22). Previous studies have shown that transcriptional factors C/EBP-α and C/EBP-δ participate in TLR-induced M1 activation, whereas C/EBP-β is involved in the polarization of macrophages to the M2 phenotype (72–74). Macrophage-specific deficiency of C/EBP-α can protect against high-fat-induced inflammation in skeletal muscle (73). C/EBP-α also acts as a tumor suppressor in the development of various cancers, including pancreatic cancer, lung cancer, acute myeloid leukemia and head and neck squamous cell carcinoma (75–78). It has been shown that C/EBPβ involves various biological functions, including a role in adipocyte differentiation, tumor progression, inflammation, and immune function (72, 79). C/EBP-δ has been shown to play a key role in adipocyte differentiation and inflammation (72, 80). More recently, the concerns have been raised about the roles of four miRs including miR-124, miR-181a, miR-155, and let-7c in regulating C/EBP in macrophage polarization.

Microglia is one of the inflammatory cells in the central nervous system (CNS), which plays a diverse role *via* polarizing into two main states, namely, classically activated M1 and alternatively activated M2 (81). Brain-specific miR-124 is expressed in microglia in the normal CNS and during experimental autoimmune encephalomyelitis (EAE). *In vivo* administration of miR-124 ameliorates both EAE symptoms and inflammation by affecting macrophages in the CNS (82). Also, the expression of miR-124 is inversely related to the activation state of microglia and macrophages in the CNS. High levels of C/EBP-α expression are associated with the activated CD45^high^ microglia. Intriguingly, transfection of macrophages with miR-124 was reported to downregulate both C/EBP-α and CD45, suggesting that the decrease in C/EBP-α corresponds to the CD45^low^ phenotype of macrophages. Moreover, transfection of macrophages with miR-124 also results in downregulation of markers and cytokines associated with pro-inflammatory M1 macrophage phenotype, including CD86, MHC class II, TNF-α, and NOS2, but the upregulation of cytokines and markers associated with anti-inflammatory M2 macrophage phenotype, including TGF-β1, Arg-1, and FIZZ1. Further study demonstrated that miR-124 controls these markers and cytokines of macrophage activation by directly suppressing the transcription factor C/EBP-α and its downstream target PU.1 (82). In addition, Yu et al. demonstrated that miR-124 promotes microglia M2 polarization and attenuates inflammatory response by directly targeting the 3′UTR of C/EBP-α for repression in intracerebral hemorrhage (81).

Besides miR-124, miR-181a is also a pivotal molecule regulating M2 mediated tumor cell migration, invasion and metastasis by targeting C/EBP-α. miR-181a is a negative regulator of M1 macrophage phenotype, which overexpression augments M2 activation and drives a transition from an M1 toward an M2 phenotype. Furthermore, luciferase assay showed that miR-181a can significantly inhibit C/EBP-α translation by targeting the 3′UTR of C/EBP-α mRNA. In addition, overexpression of miR-181a inhibits C/EBP-α protein expression (83), suggesting that miR-181a is also an important regulator of transcriptional factor C/EBP-α in macrophage polarization.

It has been shown that TAMs exhibit an M2 phenotype and are known to promote tumor proliferation. C/EBP-β is highly expressed in TAMs both *in vitro* and human tumors *in situ*. In human solid tumors, the expression of C/EBP-β protein is inversely correlated with miR-155 expression (84). Akt2 ablation can result in a decrease in miR-155 levels and an increase in C/EBP-β expression (85). Further studies have demonstrated that C/EBP-β is a direct target gene of miR-155. Induction of miR-155 significantly decreases the cytokine production in TAMs *via* inhibiting the expression of C/EBP-β at both mRNA and protein levels (84). These data suggest that miR-155 may promote the M1 polarization of macrophages *via* inhibiting C/EBP-β signaling cascade. Previous studies have demonstrated that miR-155 promotes M1 macrophage polarization through regulating STAT6 (61), suggesting that miR-155 regulates macrophage polarization by modulating different transcription factors.

In alveolar macrophages from fibrotic mouse lungs exhibiting an M2 phenotype, let-7c is upregulated when compared with those from normal mouse lungs (86–89). Overexpression of let-7c in GM-BMM (M1 macrophages) diminishes M1 phenotypic expression, while promotes macrophage polarization toward the M2 phenotype (86). These studies suggest that let-7c may be involved in pulmonary fibrosis *via* regulating alveolar macrophage polarization. Moreover, C/EBP-δ acts as an amplifier of NF-κB response, which is necessary for persistent TLR4-induced inflammatory signals (74). Banerjee et al. found that let-7c plays a crucial role in the regulation of macrophage polarization through directly targeting C/EBP-δ (86). However, C/EBP-δ may not be the only mediator for let-7c in the regulation of the macrophage polarization, because knockdown of C/EBP-δ does not duplicate all of the effects of let-7c in macrophages (86). Thus, there may be other transcription factors or cytokines to mediate let-7c effects on macrophage polarization.

### Interferon Regulatory Factors

Interferon regulatory factors were originally described as the regulators of type I IFN expression and signaling and also identified as important mediators of macrophage polarization. Several their family members have been reported in association with a specific phenotype (22, 90). IRF4 can be induced by IL-4 *via* jumonji domain-containing protein (JMJD) 3, which is important for the induction of M2 macrophage responses to helminth infection (91, 92). Also, IL-4 regulates a subset of genes associated with the M2 phenotype, including several MHC-II genes, CIITA, cytochrome P450 1B1 (CYP1B1), and IL-1 receptor antagonist gene (IL1RN), which were dysregulated in IRF4-deficient macrophages (91). IRF5 plays a significant role in regulating the polarization of adipose tissue macrophages and insulin resistance during obesity (93). LPS-induced recruitment of IRF5 promotes M1 macrophage polarization *via* equipping the cells with an IL-12^high^IL-23^high^IL-10^low^ cytokine profile (94). It has been shown that the absence of IRF9 results in profound protection from dextran sodium sulfate-induced colon inflammation (95). IRF9 is known to regulate type I interferon signaling and also plays a role in promoting M1 macrophage polarization (21, 96).

Notch signaling has been identified as an evolutionarily conserved pathway to modulate the M1/M2 polarization of macrophages. It has been reported that differentiation of TAMs depends on the transcriptional regulator of Notch signaling in a mouse mammary tumor model (97). Macrophages overexpressing miR-125a have an enhanced phagocytic activity and an inhibitory effect on tumor growth by remodeling the immune microenvironment (98). miR-125a is a downstream molecule of Notch signaling with a crucial role in promoting M1 and inhibiting M2 polarization (98). IRF4 is an important transcription factor involved in this pathway. It was found that the expression of the IRF4 protein is was markedly decreased in BMDMs transfected with miR-125a. The reporter assay showed that miR-125a reduces luciferase activity in cells transfected with reporters containing the WT 3′UTR of IRF4, which is abrogated by disruption of the proximal seed sequence (302–309 bp) in the unique seed sequence in the IRF4 3′UTR (98). Further study showed that miR-125a upregulates the M1 markers iNOS, IL-12, and TNF-α and downregulates the M2 marker mannose receptor (Mrc1) in BMDMs. IRF4 knockdown reduces Mrc1 expression and nearly reverses miR-125a ASO-mediated Mrc1 upregulation (98). These data suggest that miR-125a inhibits M2 macrophage polarization and enhances pro-inflammatory macrophage activation by targeting IRF4. Moreover, miR-125b is involved in the regulation of macrophage activation by targeting IRF4. Overexpression of miR-125b significantly enhances surface expression of costimulatory molecules and increases macrophage response to IFN-γ by inhibiting the expression IRF4 (99).

A recent study by Peng et al. showed that miR-146b strikingly inhibits M1 macrophage polarization and ameliorates the development of colitis (100). Furthermore, the expression of IRF5 was decreased in the mucosa of the colon with the treatment of miR-146b mimic. Luciferase assays demonstrated that human IRF5 is a direct target of miR-146b. Moreover, the miR-146b mimic markedly decreased the expression of IRF5 at both protein and mRNA levels in IFN-γ/LPS-induced M1 macrophages. Also, IRF5 overexpression rescued miR-146b mimic-induced inhibition of M1 macrophage polarization (100). Therefore, miR-146b inhibits M1 macrophage polarization by directly targeting IRF5.

Macrophage polarization plays a crucial role in the modulation of an angiogenesis in distal ischemic muscle. Ganta et al. demonstrated that overexpression of miR-93 induces M2-like-polarization in macrophages to promote angiogenesis (96). miR-93 modulates the expression of the immune responsive gene (IRG) 1 to promote M2 macrophage polarization. However, IRG1 is not a direct target of miR-93. Reporter assays confirmed IRF9 as a direct target of miR-93. Also, overexpression of IRF9 significantly induces IRG1-expression in macrophages (96). These data suggest that miR-93 reduces the expression of IRG1 by targeting IRF9, leading to the polarization of M2 macrophages.

### Kruppel-Like Factors

Kruppel-like factors are a subfamily of the zinc finger class of DNA-binding transcriptional regulators. Several family members of KLFs, such as KLF4 and KLF6, have been reported to play important roles in the regulation of macrophage polarization. Liao et al. found that deficiency of myeloid KLF4 results in increased pro-inflammatory cytokines in the skin and a delay in wound healing (15). KLF4 was found to cooperate with STAT6 to induce an M2 genetic program and inhibit M1 targets *via* sequestration of coactivators required for NF-κB activation (15). Goodman et al. observed that myeloid-specific deficiency of KLF6 diminishes the expression of dextran sulfate sodium-induced pro-inflammatory cytokine genes and enhances the expression of anti-inflammatory genes in colon tissues (101). KLF6 was found to promote M1 phenotype through cooperation with NF-κB and inhibit the M2 targets by suppressing the expression of PPAR-γ (102).

It is generally accepted that *Mycobacterium tuberculosis* (Mtb) interferes with M1 polarization and induces the M2 profile (103). Sahu et al. found that KLF4 upregulates Mcl-1 expression thereby repressing autophagy during Mtb infection. Furthermore, silencing of KLF4 represses arginase activity while augmenting nitrite production and the expression of iNOS during Mtb infection (104). miR-26a was validated as a negative regulator of the expression of KLF4 in Mtb-infected macrophages. The role of miR-26a in transcriptional regulation of the iNOS/arginase balance was further strengthened (104). Therefore, the miR-26a/KLF4 signaling axis is a determinant of the M1/M2 macrophage phenotype during Mtb infection. In addition, KLF6 was validated as a potential target of miR-181a. Bi et al. demonstrated that miR-181a promotes M2 macrophage polarization *via* directly targeting KLF6 (83).

### GATA Binding Protein 3

GATA binding protein 3 belongs to the GATA family of transcription factors, which are involved in the regulation of M2 macrophage polarization. GATA3 is significantly elevated in M2 macrophages. Treatment of mouse monocytic cell line with GATA3 shRNA markedly decreases the expression of M2 markers (24).

Zhong and Yi found that miR-720 inhibits breast cancer metastasis through regulating macrophage polarization in the tumor microenvironment (105). Overexpression of miR-720 inhibits the expression of genes associated with M2 phenotypes, such as CD163, IL-10, and CCL17, but has no effect on the expression of M1 marker CD86 and little effect on the production of M1 macrophage cytokines, TNF-α and IL-6 (105). These results suggest that miR-720 is important for the polarization of macrophage toward an M2 phenotype. Furthermore, the authors also revealed that GATA3 is a potential target of miR-720 involved in M2 polarization. Luciferase reporter assay revealed that GATA3 is regulated by miR-720 *via* direct binding to its 3′UTR. Also, overexpression of miR-720 results in downregulation of GATA3 expression at both mRNA and protein levels in THP-1 cells (105). Notably, GATA3 that is regulated by miR-720 inhibits M2 polarization since ectopic expression of GATA3 restores the expression of M2 marker CD163 in miR-720-overexpressed THP-1 cells. However, ectopic expression of GATA3 only partially restores the expression of other genes associated with M2 phenotypes, such as IL-10, CCL17, and Arg-1, suggesting that other downstream targets of miR-720 may also contribute to miR-720-regulated M2 polarization (105). Taken together, GATA3 is one of the miR-720 downstream targets and, at least partially, mediates the inhibitory effects of miR-720 on M2 macrophage polarization.

### Nuclear Transcription Factor-κB

In mammals, the NF-κB family of transcription factors comprises five members: RelA (p65), RelB, c-Rel, NF-κB1 (p105/p50), and NF-κB2 (p100/p52) (106). The NF-κB signaling in numerous cell types is closely related to the development of metabolic diseases and inflammatory diseases (107). NF-κB is a critical transcriptional regulator of M1 macrophage polarization induced by TLR4. TLR4 signaling activates Iκ-B kinase (IKK) to phosphorylate Iκ-B *via* myeloid differentiation primary response gene 88 (MyD88)-dependent and MyD88-independent [TIR domain-containing adapter-inducing IFN-β (TRIF)-dependent] pathways. Moreover, activation of the NF-κB is initiated by Iκ-B phosphorylation in response to microenvironmental stimuli. After the phosphorylation of Iκ-B and subsequent degradation, NF-κB translocates to the nucleus and controls the expression of various target genes by binding to specific DNA sequences (107, 108). Activation of NF-κB promotes M1 macrophage polarization by regulating the expression of pro-inflammatory cytokines.

miR-210 plays an important role in both inflammation and immunity. It inhibits pro-inflammatory cytokine expression and the NF-κB pathway in osteoarthritis (109). Qi et al. found that miR-210 functions as a negative regulator of LPS/TLR4 signaling (110). Overexpression of miR-210 suppresses the TLR4-induced expression of pro-inflammatory cytokines TNF-α and IL-6 *via* targeting NF-κB in macrophages. Furthermore, the miR-210 mimic can inhibit NF-κB activation by targeting molecules downstream of IKK-β in LPS-stimulated macrophages. In addition, Zhang and his colleagues found that miR-210 mimic suppresses the activation of NF-κB *via* increasing the Iκ-Bα level and reducing the p65 expression in LPS-treated cells (109). These data suggest that miR-210 acts as a key regulator to inhibit M1 macrophage polarization, which may provide a therapeutic target for inflammatory diseases.

### c-MYC

c-MYC is a critical transcription factor in the regulation of alternative macrophage activation and TAM biology (111, 112). It acts as a transcriptional activator to regulate the expression of target genes by binding to enhancer box sequences (E-boxes) in the promoter region. It has been confirmed that c-MYC can directly regulate a significant set of M2-associated gene expression (ALOX15, Mrc1, and SCARB1). Also, c-MYC promotes M2 macrophage polarization by upregulating the IL-4 signaling mediators STAT6 and PPAR-γ (111).

miR-26 family, including miR-26a and miR-26b, was reported to be closely linked to the pathogenesis of esophageal squamous cell carcinoma (ESCC). Li et al. showed that miR-26a and miR-26b expression was significantly decreased in the majority of ESCC tissues (113). Both miR-26a and -26b inhibit ESCC cell proliferation *via* directly targeting the MYC binding protein (MYCBP) 3′UTRs for repression. MYCBP binds to the N-terminal region of c-MYC and enhances its ability to activate E box-dependent transcription. Moreover, upregulation of miR-26a and -26b results in a decreased expression of most c-MYC target genes, suggesting that miR-26 family can inhibit ESCC cell proliferation *via* suppression of MYCBP and subsequent inhibition of the c-MYC pathway (113). It is well received that TAMs play a crucial role in tumor growth, invasion, angiogenesis, and metastasis formation. Given that c-MYC is a key transcription factor in the regulation of TAM biology, miR-26 family members may act as tumor suppressors by suppressing the c-MYC pathway and decrease TAMs in ESCC tissues.

## Conclusion

During the past few years, significant progress has been made to unravel how transcription factors define macrophage identity and control macrophage polarization during homeostasis or in challenging situations (23). Recently, miRNAs have been found to act as upstream regulators of transcription factors involved in the regulation of macrophage polarization (PPARs, STATs, C/EBP, IRFs, KLFs, GATA3, NF-κB, and c-MYC). The biological role of miRNA-transcription factor networks for macrophage polarization involves the development of multiple diseases. An outline of the role of miRNAs in the transcriptional regulation of macrophage polarization provides insights into their regulation of inflammation, immune response and tumor. However, the mechanism for miRNAs to regulate macrophage polarization *via* transcription factors appears complicated. For example, Wang et al. demonstrated that miR-21 promotes M1 and inhibits M2 peritoneal macrophage polarization by directly targeting STAT3 (66). However, it has been also shown that miR-21 inhibits the pro-inflammatory macrophage polarization and enhances the anti-inflammatory macrophage phenotype in other macrophage cell lines (67, 68). The reasons for this discrepancy still remain uncertain. Since miRNAs are specific for different tissues and cell types, it is possible that miR-21 exerts different effects on macrophage polarization depending on cell types and *in vivo* microenvironment. We summarized the similarities and differences in miRNAs that regulate macrophage polarization in mouse and human (Table 2). Furthermore, a single miRNA, such as miR-181a and miR-155, is known to target multiple transcription factors involved in macrophage polarization. In addition, one transcription factor in macrophage polarization also cooperatively binds to various miRNAs, particularly in the cancer microenvironment.

**Table 2:** The effects of microRNAs (miRNAs) on macrophage polarization in mouse and human.

miRNAs	Regulation of macrophage phenotype			
	Mouse	Reference	Human	Reference
miR-9	–		Suppresses M1	(37)
miR-27a	Promotes M1, suppresses M2	(40, 71)	Promotes M1, suppresses M2	(71)
miR-27b	Promotes M1, suppresses M2	(43)	Promotes M1, suppresses M2	(43)
miR-130a	–		Promotes M1, suppresses M2	(45)
miR-130b	Promotes M1, suppresses M2	(46)	–	
miR-146a	Suppresses M1, promotes M2	(48, 53)	Suppresses M1, promotes M2	(51)
miR-146b	Suppresses M1, promotes M2	(100)	Suppresses M1, promotes M2	(54)
miR-155	Promotes M1, suppresses M2	(60, 64)	Promotes M1, suppresses M2	(61–63)
miR-21	Promotes M1, suppresses M2	(66)	–	
	Suppresses M1, promotes M2	(67, 68)	–	
miR-222-3p	–		Promotes M2	(69)
miR-19a-3p	Suppresses M2	(70)	Suppresses M2	(70)
miR-23a	Suppresses M2	(71)	–	
miR-124	Suppresses M1, promotes M2	(81, 82)	–	
miR-181a	Suppresses M1, promotes M2	(83)	Suppresses M1, promotes M2	(83)
let-7c	Suppresses M1, promotes M2	(86)	–	
miR-125a	Promotes M1, suppresses M2	(98)	–	
miR-125b	Promotes M1, suppresses M2	(99)	–	
miR-93	Suppresses M1, promotes M2	(96)	–	
miR-26a	Promotes M1, suppresses M2	(104)	Suppresses M2	(111, 113)
miR-26b	–		Suppresses M2	(111, 113)
miR-720	–		Suppresses M2	(105)
miR-210	Suppresses M1	(109, 110)	–	

*–, Not determined*.

In addition to the canonical function of binding to their target messenger RNAs, tumor-secreted miR-21 and miR-29a may function through other mechanisms, for example, by binding as ligands to receptors of the TLR family, murine TLR7 and human TLR8 in immune cells and triggering a TLR-mediated prometastatic inflammatory response, to regulate macrophage polarization (65). Lehmann et al. also found that let-7b can act as a potent activator of TLR7 signaling in neurons and this activation may induce neurodegeneration (114). The GU-rich motif (GUUG for miR-21, GGUU for miR-29a, and GUUGUGU for let-7b) is essential for the miRNA-TLR recognition. This may be an important pathway to modulate macrophage polarization. However, whether these miRNAs with “GU” rich sequence can directly bind to transcription factors and regulate macrophage polarization needs further investigation.

The extent of macrophage infiltration serves as an important diagnostic and prognostic biomarker in many human cancers (115). An increased number of CD68^+^ (a typical mark of TAMs) macrophages was strongly associated with shortened survival in patients with classic Hodgkin’s lymphoma, suggesting a new biomarker for risk stratification (116). High macrophage density at the tumor front positively influences prognosis in colorectal cancer (117). In addition, several lines of evidence indicate that new strategies manipulating TAMs represent an active research area for improving anti-tumor therapies (118, 119). TAM depletion was accompanied by significant inhibition of tumor growth in both syngeneic and xenogenic tumor models. Treatment of tumor-bearing mice with clodrolip in combination with anti-VEGF single chain fragment antibodies significantly enhanced the depletion of TAMs and resulted in drastic tumor growth inhibition (120). In mice harboring glioblastoma multiforme xenografts, treatment with pexidartinib (PLX3397) augmented tumor responsiveness to radiotherapy by reducing the recruitment of bone marrow-derived TAMs (121). However, the extent to which their effects on macrophages explain their clinical efficacy still remains to be defined. Understanding the molecular mechanisms for the miRNA-transcription factor networks to regulate macrophages helps provide a basis for macrophage-centered diagnostic and therapeutic strategies.

In view of the importance and multifaceted contribution of macrophage polarization in many physiological and pathological conditions including infection, inflammation, immunity, regeneration, and cancer, more research is required for a better understanding of how multiple target miRNAs are required to modulate transcription factors and how they cooperatively balance macrophage polarization in various diseases.

## Author Contributions

G-JZ conceived and designed the article. HL and TJ surveyed the literature and wrote the article. M-QL and X-LZ surveyed the literature and provided suggestions. All the authors have approved the manuscript for submission.

## Conflict of Interest Statement

The authors declare that there is no conflict of interests regarding the publication of this paper.
